# Alcohol consumption and survival after breast cancer diagnosis in Japanese women: A prospective patient cohort study

**DOI:** 10.1371/journal.pone.0224797

**Published:** 2019-11-13

**Authors:** Yuko Minami, Seiki Kanemura, Masaaki Kawai, Yoshikazu Nishino, Hiroshi Tada, Minoru Miyashita, Takanori Ishida, Yoichiro Kakugawa

**Affiliations:** 1 Division of Community Health, Tohoku University Graduate School of Medicine, Sendai, Miyagi, Japan; 2 Division of Cancer Epidemiology and Prevention, Miyagi Cancer Center Research Institute, Natori, Miyagi, Japan; 3 Center for Preventive Medicine, Osaki Citizen Hospital, Osaki, Miyagi, Japan; 4 Department of Breast Surgery, Miyagi Cancer Center Hospital, Natori, Miyagi, Japan; 5 Department of Epidemiology and Public Health, Kanazawa Medical University, 1–1 Daigaku, Uchinada, Kahoku, Ishikawa, Japan; 6 Department of Breast and Endocrine Surgical Oncology, Tohoku University Graduate School of Medicine, Sendai, Miyagi, Japan; 7 Department of Surgery, Japanese Red Cross Sendai Hospital, Sendai, Miyagi, Japan; West Virginia University, UNITED STATES

## Abstract

**Background:**

It is unclear whether alcohol consumption may impact survival after breast cancer diagnosis. To clarify the association between pretreatment alcohol consumption and survival in breast cancer patients, a prospective patient cohort study was conducted.

**Methods:**

The cohort comprised 1,420 breast cancer patients diagnosed during 1997–2013 at a single institute in Japan. Alcohol drinking and other lifestyle factors were assessed by questionnaire survey at the initial admission. The patients were followed until December 31, 2016. The crude associations of pretreatment alcohol intake with survival were evaluated by Kaplan-Meier analysis. The Cox proportional hazards model was used to estimate hazard ratios (HRs) and 95% confidence intervals (CIs) controlled by confounders.

**Results:**

During a median follow-up period of 8.6 years, 261 all-cause and 193 breast cancer-specific deaths were documented. Survival curves showed that ever-drinkers tended to have better survival than never-drinkers (breast cancer-specific survival, log-rank p = 0.0381). Better survival was also observed for light drinkers with an intake of <5.0 g per day. In the Cox model, ever-drinking was associated with a decreased risk of all-cause (HR: 0.75; 95% CI: 0.54–1.05) and breast cancer-specific death (HR: 0.68; 95% CI: 0.46–0.99). Light drinkers had a lower risk of breast cancer-specific death (frequency of drinking, HR = 0.57 for occasional or 1–2 times per week and 0.72 for 3–7 times per week; amount of alcohol consumed per day, HR = 0.57 for <5.0 g and 0.68 for ≥5.0 g compared with never-drinking). In terms of hormone receptor status, a significantly decreased risk of death associated with ever-drinking was observed among women with receptor-negative cancer (ER-/PR-, HR = 0.41; 95% CI: 0.20–0.84 for breast cancer-specific death).

**Conclusions:**

Pretreatment, i.e., pre-diagnosis alcohol consumption is unlikely to have an adverse effect on survival after breast cancer diagnosis. Light alcohol consumption may have a beneficial effect on patient survival.

## Introduction

Epidemiologic studies have shown that alcohol consumption is associated with an increased risk of breast cancer, and it is now regarded as an established risk factor for the disease [1, 2]. Data from some recent Japanese studies have also provided support for this [3, 4]. On the other hand, alcohol consumption may also impact survival after breast cancer diagnosis. Owing to recent advances in treatment, breast cancer survival rates have been improving [5, 6, 7]. Additionally, breast cancer incidence has been continuously increasing in Japan [8]; therefore, the population of survivors continues to grow. Whether modifiable lifestyle factors such as alcohol consumption contribute to improved or adverse survival may be a concern for breast cancer patients [9]. However, previous studies have yielded conflicting results: some have shown that alcohol consumption is associated with lower survival [10–12], whereas others have reported no such association [13–19] or even an improved survival effect [20, 21]. With regard to pre- and post-diagnosis alcohol consumption, a meta-analysis including 11 studies showed that pre-diagnosis consumption was associated with better overall survival, whereas post-diagnosis consumption had no association with overall survival [22]. Another meta-analysis including 25 studies concluded that neither pre- nor post-diagnosis alcohol consumption was associated with breast cancer-specific mortality [23]. Pooled analyses including three breast cancer cohorts have evaluated the mortality risk stratified by estrogen receptor (ER) status [22]. In one cohort comprising multiple studies (the BCAC study), improved overall survival associated with pre-diagnosis consumption was found among women with ER+ cancer [22]. Another cohort study (the SEARCH study) showed that post-diagnosis consumption was associated with improved overall and breast cancer-specific survival among women with ER- cancer [22]. These studies appear to provide useful information when considering the impact of lifestyle on survival of breast cancer patients; however, there were differences in outcome measures (overall or breast cancer-specific survival, etc.) among the individual studies. Data for alcohol-related mortality risk in relation to hormone receptor status have been limited [13, 21, 22]. Furthermore, most of the previous studies have been conducted in Western countries. To our knowledge, the mortality risk associated with alcohol consumption has never been investigated among Asian breast cancer patients, including those from Japan. Asian women have a low alcohol intake relative to that of women in Western countries [24].

In order to clarify the association between alcohol consumption and survival after breast cancer diagnosis, we conducted a hospital-based prospective patient cohort study. Data on alcohol-related measures and clinical information for breast cancer patients were obtained from a questionnaire survey at their first admission and from a hospital-based cancer registry at a single institute in Japan, and a long-term follow-up survey was conducted. In this study, we evaluated the risk of all-cause and breast cancer-specific death stratified by hormone receptor status as well as the risk among the patients overall.

## Methods

This study was approved by the Management Committee of the Miyagi Cancer Center (December 9, 1996) and the Ethics Committee of the Miyagi Cancer Center (Approval Number 23–7, May 20, 2011) and conducted in accordance with the principles specified in the Declaration of Helsinki. The questionnaire survey in connection with the present study was started with the approval of the Management Committee. The Ethics Committee independently reviewed the protocol of the present study that used the questionnaire data and cancer registry data. The purpose of the study was stated on the cover page of the questionnaire. We considered the return of self-administered questionnaires signed by the patients to imply their consent to participate in the study.

### Study subjects

Between January 1997 and December 2013, 1,570 first-admitted female patients aged 20 years or over without a history of cancer were diagnosed as having breast cancer at the Miyagi Cancer Center Hospital (MCCH). At the time of admission, these patients were requested to complete a self-administered questionnaire including history of alcohol drinking. After diagnosis, the patients were registered to the hospital-based cancer registry and followed up. Details of the questionnaire survey and cancer registry have already been described elsewhere [25, 26]. Among the patients, 1,488 (94.8%) completed the questionnaire. After excluding 20 patients who had cancers other than breast cancer at the time of breast cancer diagnosis, the remaining 1,468 patients were identified for the patient cohort.

The questionnaire covered demographic characteristics, personal and family histories of cancer and other diseases, current height and weight, general lifestyle factors including alcohol intake, dietary history, cigarette smoking, physical activity, and menstrual and reproductive histories, and referral status. We obtained data on alcohol consumption and other lifestyle factors from this questionnaire. Dietary history and alcohol intake were evaluated using a food frequency questionnaire (FFQ). The FFQ has been validated in general population, whose residential area was roughly the same as that of our study subjects [27].

Clinical information including tumor stage based on the UICC TNM classification, and initial adjuvant treatment was obtained from the MCCH cancer registry. Information on estrogen/progesterone receptor (ER/PR) expression were extracted from medical records.

### Assessment of exposure and follow up

At the MCCH, in principle, detailed diagnostic tests and initial therapy are initiated after admission. Therefore, exposure data (alcohol consumption) collected by the questionnaire survey were considered to be pretreatment or pre-diagnosis data.

For assessment of alcohol intake, the FFQ asked initially if the study subjects were never, past, or current drinkers. Past or current drinkers were asked to state age at start of drinking, frequency of drinking per week, and the types of alcohol beverages consumed [Japanese *sake*, Japanese spirits (*shochu*), beer, whisky, wine and others]. For each type of alcohol beverage consumed, they were also asked to state the volume drunk after conversion into the Japanese *sake* equivalent by reference to a conversion table (S1 and S2 Files). One unit (180 ml) of Japanese *sake* contains 22.8 g of ethanol (alcohol). The amount of alcohol consumed per day was calculated as: (total amount of alcohol consumed per occasion (g)) x (frequency of drinking per week) / 7 [27].

The exposure variables related to alcohol consumption included history of alcohol drinking (never, ever, past, current), frequency of drinking per week (never, occasional or 1–2 times, 3–7 times), age at start of drinking (never, ≤20 years, ≥21 years), and amount of alcohol consumed per day (never, <5.0 g, ≥5.0 g). Although, in our previous case-control and cohort studies of breast cancer, the quantitative exposure variable had been classified based on finer categories [28, 29], the present study used the mid-point for variable categorization because of the limited number of patients. If necessary, analysis based on the finer categories was also performed. Patients for whom data on history of alcohol drinking were missing (n = 48) were excluded from the subsequent analysis, leaving a total of 1420 patients as the analyzed subjects.

Follow-up of patients was performed by reference to the MCCH cancer registry up to December 2016. The registry conducted active follow-up by accessing hospital visit records, resident registration cards and permanent domicile data. For deceased patients, information on the date and cause of death was obtained with permission from the Ministry of Justice. As a general rule for this registry, each individual follow-up ends in the 11th year after diagnosis [30]. Therefore, information on vital status at 11 years was available for the patients diagnosed during 1997–2005. For the patients diagnosed after 2006, vital status as of December 2016 was obtained. During the follow-up period, 3 patients (0.2%) were lost to follow-up, and these were treated as censored cases.

### Statistical analysis

The end point of our analysis was all-cause death and breast cancer-specific death according to the International Classification of Disease for Oncology, Tenth Edition (ICD-10). Survival time was calculated for each patient from the date of diagnosis to the date of death or the end of follow-up.

To investigate the risk of all-cause and breast cancer-specific death, Kaplan-Meier survival analysis and Cox proportional hazards model were used. The crude associations of exposures with survival were evaluated by Kaplan-Meier analysis. The Cox proportional hazards model was used to estimate hazard ratios (HRs) and 95% confidence intervals (CIs) controlled by confounders. We considered the following variables to be potential confounders: age, year of diagnosis, tumor stage (0-I, II, III, IV), hormone receptor status (ER+ or PR+, ER-/PR-), menopausal status (premenopausal, postmenopausal, missing), radiation therapy (no, yes), chemotherapy (no, yes), endocrine therapy (no, yes), family history of breast cancer in father, mother, brother or sister (no, yes), body mass index (BMI) (<21.1, 21.1-<23.3, 23.3-<26.0, ≥26.0, missing), physical activity (almost no, more than one hour per week, missing), occupation (housewife, other), referral status (from screening, other), and comorbidities (no, yes). Comorbidities included hypertension, ischemic heart disease, stroke and diabetes mellitus. Reproductive factors including menarche and parity, history of smoking and folate intake were also considered as confounders. Our previous studies had revealed associations of reproductive factors, BMI and smoking with patient survival [25, 26, 30]. Folate intake may modify the association of alcohol intake with the development of breast cancer [28, 31]. Missing values for confounders were treated as an additional variable category and included in the model.

For analysis using the Cox model, we first evaluated HRs according to exposure among the patients overall. Second, separate analyses were conducted by dividing the patients according to hormone receptor status. Third, some stratified analyses were performed to examine whether the association of alcohol intake with survival differed in relation to selected factors including menopausal status, BMI, and folate intake. Results were regarded as significant if the two-sided P values were <0.05. For each analysis, the proportional hazards assumption was assessed based on log–log plots of survival and testing time dependent cross-product terms of the survival time and exposures. The cross-product terms were not significant in any of the models, indicating no significant violation of the assumptions. Statistical analyses were performed using the SAS software package (version 9.4; SAS Institute) and JMP software (pro 14, SAS Institute).

## Results

During a median follow-up period of 8.6 years (11,180.9 person-years), 261 all-cause and 193 breast cancer-specific deaths were observed. Table 1 shows the characteristics of the patients at the baseline. Among 1420 study subjects, 405 (28.5%) were ever-drinkers. Current drinkers tended to be ever-smokers, younger and premenopausal, to have a lower BMI, and to have fewer comorbidities than never-drinkers. With regard to hormone receptor status, more than 60% of current drinkers had ER+/PR+ cancer. Among past- and current drinkers, none died of vascular diseases.

**Table 1:** Characteristics of the study subjects.

Characteristics	Tota (n = 1420)^a^	Alcohol drinking at diagnosis			
		Never (n = 1015)	Past (n = 48)	Current (n = 357)	p^d^
Person-years	11180.9	7922.7	348.6	2909.6	
All-cause death, n	261	206	8	47	
Cause of death, n					
Vascular diseases	15	15	0	0	
Other cancers	21	17	0	4	
Others	32	24	2	6	
Breast cancer	193	150	6	37	
Age (years)					
Mean	57.2	59.3	56.1	51.4	
S.D.	12.4	12.4	12.0	10.5	<0.001
Year of diagnosis (%)					
1997–2005	44.5	46.3	31.3	41.2	
2006–2013	55.5	53.7	68.7	58.8	0.04
Stage (%)					
0-I	50.4	49.9	47.9	52.4	
II	30.9	31.0	29.2	30.5	
III	11.1	11.1	10.4	10.9	
IV	6.4	6.6	10.4	5.3	
Missing	1.3	1.4	2.1	0.8	0.92
Hormone receptor (%)					
ER+/PR+	55.3	53.3	47.9	62.2	
ER+/PR-	12.1	13.3	16.7	8.1	
ER-/PR+	1.6	1.8	0.0	1.1	
ER-/PR-	24.0	23.8	27.1	24.1	
Missing	7.0	7.8	8.3	4.5	0.03
Referral status (%)					
From screening	18.5	18.7	12.5	18.8	
Other	81.5	81.3	87.5	81.2	0.55
Radiation (%)					
No	59.6	62.2	56.3	52.9	
Yes	40.4	37.8	43.7	47.1	0.01
Chemotherapy (%)					
No	68.8	70.1	60.4	66.1	
Yes	31.2	29.9	39.6	33.9	0.16
Endocrine therapy (%)					
No	56.0	55.7	58.3	56.6	
Yes	44.0	44.3	41.7	43.4	0.90
Family history of breast cancer in father, mother, brother or sister (%)					
No	90.0	89.8	95.8	89.9	
Yes	10.0	10.2	4.2	10.1	0.39
Age at menarche (years, %)					
≤12	32.3	28.6	33.3	42.9	
13	22.3	21.8	16.7	24.4	
14	20.4	20.1	25.0	20.5	
≥15	20.9	24.5	22.9	10.1	
Missing	4.2	5.0	2.1	2.2	<0.001
Parity history (%)					
Nulliparous	10.8	9.8	18.7	12.6	
Parous	85.8	86.3	77.1	85.4	
Missing	3.4	3.9	4.2	2.0	0.08
Menopausal status (%)					
Premenopausal	38.0	31.7	31.2	56.9	
Postmenopausal	57.1	63.8	62.5	37.3	
Missing	4.9	4.4	6.3	5.9	<0.001
Smoking (%)					
Never	80.3	87.2	54.2	64.1	
Ever	17.9	11.1	43.8	33.6	
Missing	1.8	1.7	2.1	2.2	<0.001
Physical activity (%)					
Almost no	50.3	49.4	45.8	53.5	
More than one hour per week	43.6	43.6	45.8	43.4	
Missing	6.1	7.1	8.3	3.1	0.08
BMI (kg/m^2^, %)					
<21.1	25.1	21.7	25.0	34.7	
21.1-<23.3	23.9	22.9	20.8	27.2	
23.3-<26.0	25.2	26.1	25.0	22.7	
≥26.0	24.7	28.2	29.2	14.3	
Missing	1.1	1.2	0.0	1.1	<0.001
Comorbidities (%)^b^					
No	76.0	73.4	68.8	84.3	
Yes	24.0	26.6	31.2	15.7	<0.001
Occupation (%)					
Housewife	19.4	20.6	16.7	16.3	
Other	68.2	65.0	70.8	77.0	
Missing	12.4	14.4	12.5	6.7	<0.001
Dietary intake (mean ± SD)					
Folate intake (μg per day)^c^	211.2 ± 71.9	216.1 ± 74.2	199.7 ± 78.0	199.0 ± 62.5	<0.001
Energy intake (kcal per day)	1193.7 ± 272.6	1192.3 ± 272.5	1133.4 ± 350.8	1205.7 ± 260.3	0.22

Abbreviations: BMI, body mass index; ER, estrogen receptor; PR, progesterone receptor.

^a^Patients for whom data on history of alcohol drinkin were missing (n = 48) have been excluded.

^b^Comorbidities include hypertension / ischemic heart disease / stroke / diabetes mellitus.

^c^Energy-adjusted intake.

^d^Chi-square test or anaysis of variance for comparing frequencies and means among never, past, and current drinkers.

**Fig 1:**
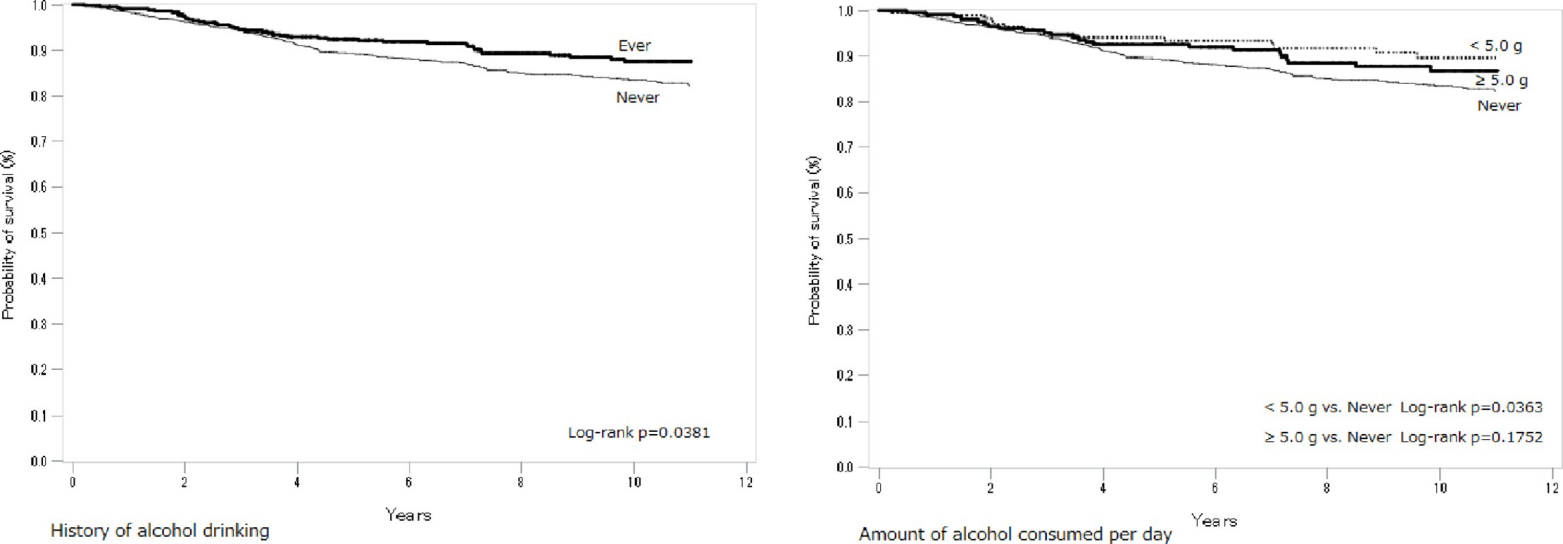
Probability of breast cancer-specific survival in relation to history of drinking and amount of alcohol consumed per day among patients overall.

**Fig 2:**
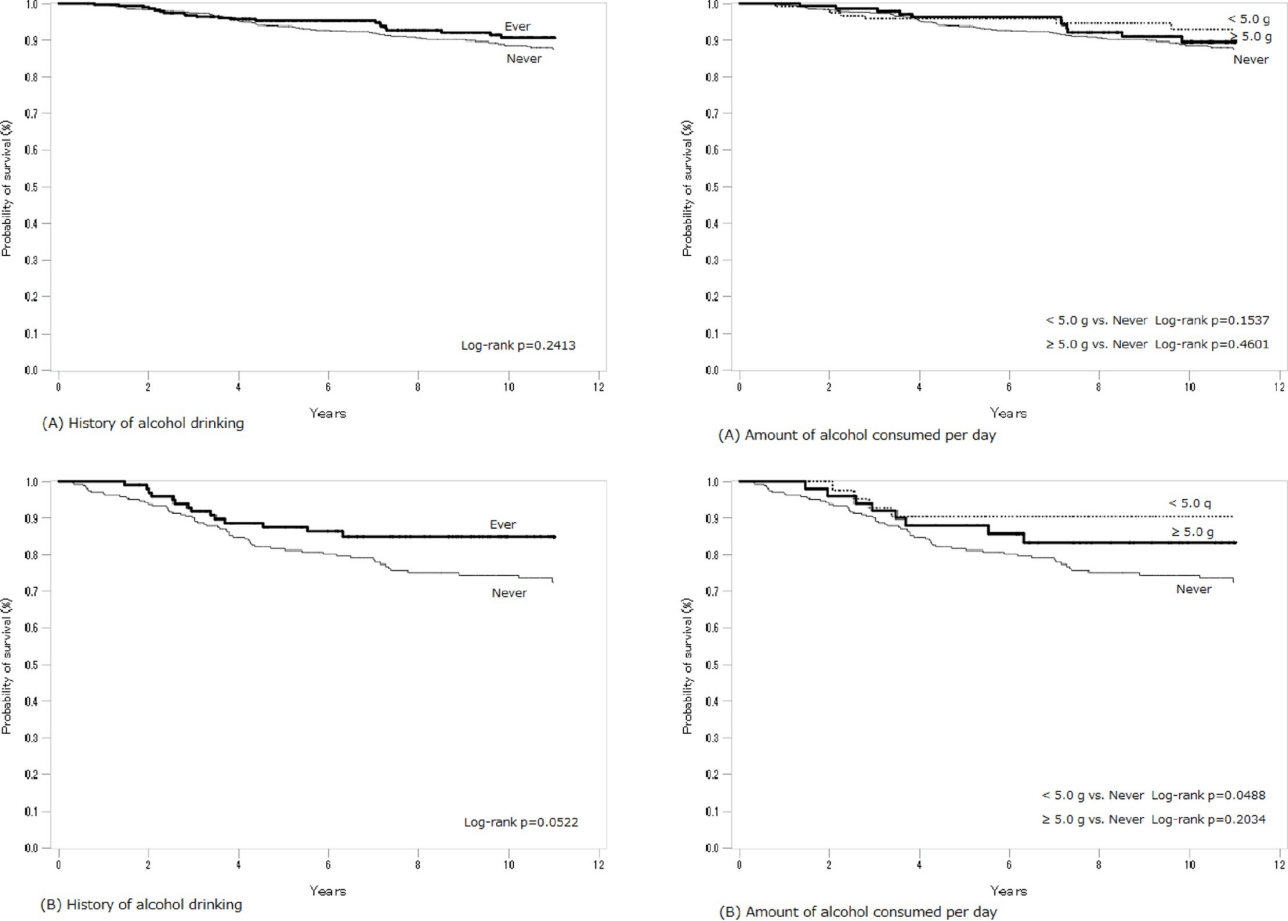
Probability of breast cancer-specific survival in relation to history of drinking and amount of alcohol consumed per day according to hormone receptor status. (A) Receptor positive cancer (ER+ or PR+). (B) Receptor negative cancer (ER-/PR-).

Fig 1 shows the Kaplan-Meier curves for breast cancer-specific survival among the patients overall. The analysis was performed for two selected alcohol-related measures (history of alcohol drinking and amount of alcohol consumed per day). The amount of alcohol consumed per day is considered an indicator of alcohol consumption level for each subject. Ever-drinkers tended to have better survival than never-drinkers (log-rank p = 0.0381). The highest survival was seen in light drinkers (<5.0 g/day), with heavier drinkers having survival intermediate between light drinkers and non-drinkers. Fig 2 shows the survival curves according to hormone receptor status. Although the association of history of alcohol drinking with survival was uncertain among patients with ER+ or PR+ cancer, ever-drinkers with ER-/PR- cancer tended to have better survival (log-rank p = 0.0522). As a whole, the Kaplan-Meier curves indicated better survival among ever-drinkers. However, most of log-rank test showed mild- or non-significance possibly due to the small number of events (Fig 1 and Fig 2). The association between alcohol consumption and the risk of breast cancer-specific death may be unclear in the standard Kaplan-Meier analysis [32]. Therefore, to further investigate this association, we attempted a bootstrap approach [32–34]. For example, the number of times with p<0.05 in 100 bootstrap random samples was 63 for the p-value (ever- vs. never-drinking) in Fig 1. The variability in survival curves exists according to history of drinking and consequently, the log-rank p may not have been fully validated. These results based on the bootstrap approach may indicate that statistical biases such as confounders should be appropriately controlled for in the subsequent analyses using the Cox model [33].

Table 2 shows the association of alcohol consumption with the risk of all-cause and breast cancer-specific death among the patients overall based on the Cox models. In the analysis based on quantitative variables, we estimated the risk using finer categorization, as well as by mid-point categorization. In the multivariate-adjusted 1 model, only clinical factors were controlled for. Other confounders were considered in the multivariate-adjusted 2 model. Although the additional adjustment for other confounders changed the magnitude of risk slightly, the direction in risk was similar between the two models. The analyses on history of drinking and other alcohol-related measures indicated that alcohol consumption was inversely associated with the risk of all-cause and breast cancer-specific death. These inverse associations were clearer for the risk of breast cancer-specific death. Ever-drinkers had a significantly lower risk of breast cancer-specific death compared with never-drinkers (HR = 0.68, 95% CI: 0.46–0.99 in multivariate-adjusted 2, p = 0.047). On the basis of quantitative variables, light drinkers tended to have a lower risk of breast cancer-specific death (occasional intake or 1–2 times per week; HR = 0.57, p = 0.048; amount of alcohol consumed per day<5.0 g; HR = 0.57, p = 0.050). The analysis based on finer categorization showed that heavy drinkers (5–7 times per week) tended to have a higher risk of all-cause death (HR = 1.04) than light-to-moderate drinkers (1–2 times, HR = 0.55; 3–4 times, HR = 0.52), suggesting a non-linear association between alcohol consumption and all-cause mortality.

**Table 2:** Hazard ratio of all-cause and breast cancer-specific death associated with alcohol drinking among overall women.

Factor	Patients	Person-years	All-cause death			Breast cancer-specific death		
			Deaths	Multivariate-adjusted 1^a^	Multivariate-adjusted 2^b^	Deaths	Multivariate-adjusted 1^a^	Multivariate-adjusted 2^b^
				HR (95% CI)	HR (95% CI)		HR (95% CI)	HR (95% CI)
History of alcohol drinking								
Never	1015	7922.7	206	1 (Reference)	1 (Reference)	150	1 (Reference)	1 (Reference)
Ever (Current/past)	405	3258.2	55	0.78 (0.57–1.06)	0.75 (0.54–1.05)	43	0.67 (0.47–0.96)	0.68 (0.46–0.99)
Past	48	348.6	8	0.80 (0.39–1.64)	0.70 (0.32–1.52)	6	0.57 (0.24–1.32)	0.53 (0.21–1.33)
Current	357	2909.6	47	0.77 (0.55–1.08)	0.76 (0.53–1.08)	37	0.70 (0.48–1.01)	0.70 (0.47–1.04)
Age at start of drinking (years)								
≥ 21	173	1418.3	25	0.74 (0.49–1.13)	0.73 (0.47–1.13)	20	0.72 (0.45–1.16)	0.74 (0.45–1.21)
≤ 20	199	1575.6	27	0.89 (0.58–1.37)	0.87 (0.55–1.36)	20	0.65 (0.40–1.07)	0.65 (0.39–1.10)
p for trend				0.33	0.31		0.04	0.07
(Finer categorization)								
> 25	111	901.1	15	0.79 (0.47–1.35)	0.79 (0.45–1.36)	12	0.91 (0.50–1.64)	0.87 (0.47–1.63)
≥21 - ≤ 25	62	517.2	10	0.67 (0.35–1.28)	0.66 (0.34–1.28)	8	0.55 (0.27–1.14)	0.59 (0.28–1.25)
≤ 20	199	1575.6	27	0.89 (0.58–1.37)	0.86 (0.55–1.36)	20	0.65 (0.39–1.06)	0.65 (0.39–1.10)
p for trend				0.31	0.29		0.03	0.05
Frequency of drinking (times)								
Occasional or 1–2 per week	187	1524.5	22	0.67 (0.42–1.05)	0.70 (0.44–1.13)	16	0.52 (0.31–0.89)	0.57 (0.33–0.99)
3–7 per week	198	1586.7	28	0.83 (0.55–1.24)	0.75 (0.48–1.17)	23	0.76 (0.48–1.19)	0.72 (0.43–1.18)
p for trend				0.17	0.12		0.07	0.08
(Finer categorization)								
Occasional	96	800.0	15	0.77 (0.45–1.32)	0.81 (0.46–1.41)	10	0.53 (0.27–1.03)	0.58 (0.29–1.14)
1–2 per week	91	724.5	7	0.52 (0.24–1.12)	0.55 (0.25–1.21)	6	0.51 (0.22–1.16)	0.57 (0.24–1.33)
3–4 per week	94	775.9	11	0.58 (0.31–1.08)	0.52 (0.27–1.00)	11	0.62 (0.33–1.16)	0.58 (0.29–1.15)
5–7 per week	104	810.8	17	1.13 (0.68–1.88)	1.04 (0.60–1.80)	12	0.95 (0.52–1.72)	0.90 (0.47–1.71)
p for trend				0.28	0.20		0.13	0.15
Amount of alcohol consumed per day (g)								
< 5.0	171	1395.9	20	0.65 (0.40–1.04)	0.68 (0.42–1.11)	15	0.52 (0.30–0.89)	0.57 (0.32–1.00)
≥ 5.0	204	1649.4	28	0.81 (0.54–1.22)	0.72 (0.46–1.12)	23	0.73 (0.47–1.15)	0.68 (0.41–1.12)
p for trend				0.14	0.08		0.05	0.06
(Finer categorization)								
< 5.0	171	1395.9	20	0.65 (0.40–1.04)	0.68 (0.42–1.11)	15	0.52 (0.30–0.89)	0.57 (0.32–1.00)
≥5.0 - < 15.0	115	937.8	15	0.69 (0.40–1.18)	0.61 (0.35–1.08)	14	0.69 (0.39–1.21)	0.66 (0.35–1.22)
≥ 15	89	711.6	13	1.01 (0.57–1.79)	0.90 (0.49–1.66)	9	0.82 (0.41–1.62)	0.71 (0.34–1.48)
p for trend				0.26	0.15		0.09	0.08

Abbreviations: HR, hazard ratio; CI, confidence interval.

^a^Adjusted by age, year of diagnosis, stage (0-I, II, III, IV, missing), hormone receptor (ER+ or PR+, ER-/PR-, missing), and menopausal status (premenopausal, postmenopausal, missing).

^b^Adjusted by age, year of diagnosis, stage (0-I, II, III, IV, missing), hormone receptor (ER+ or PR+, ER-/PR-, missing), referral status (from screening, other), radiation therapy (no, yes), chemotherapy (no, yes), endocrine therapy (no, yes), family history of breast cancer in parents or siblings (no, yes), smoking (ever, never, missing), BMI (<21.1, 21.1-<23.3, 23.3-<26.0, 26.0-, missing), physical activity (almost no, more than one hour per week, missing), comorbidities (no, yes), occupation (housewife, other, missing), age at menarche (≤12, 13, 14, 15<, missing), parity history (nulliparous, parous, missing), menopausal status (premenopausal, postmenopausal, missing), and intake of folate (continuous) and energy (continuous).

Table 3 shows the risk of all-cause and breast cancer-specific death stratified by hormone receptor status (ER+ or PR-, ER-/PR-). Among the patients with ER+ or PR+ cancer, alcohol consumption tended to reduce the risk of all-cause and breast cancer-specific death; however, statistical tests failed to demonstrate significance. On the other hand, ever-drinking was significantly associated with a decreased risk of all-cause and breast cancer-specific death among the patients with ER-/PR- cancer (all-cause death, HR = 0.49, p = 0.03; breast cancer-specific death, HR = 0.41, p = 0.01 in comparison to never-drinking). Kaplan-Meier survival curves also indicated better survival among alcohol drinkers with ER-/PR- cancer, as shown in Fig 2. However, the interaction term for assessing heterogeneity in risk across hormone receptor status was statistically insignificant. Among patients with ER-/PR- cancer, an early start to alcohol drinking tended to be associated with a lower risk of breast cancer-specific death (HR = 0.44 for ≤20 years, p for trend = 0.04), although the analysis was based on a limited number of patients. Inverse associations were also observed for frequency of drinking (p for trend = 0.04) and the amount of alcohol consumed per day (p for trend = 0.03). Furthermore, we evaluated the mortality risk stratified by ER status (Table 4), as some previous studies have focused on ER status [13, 21, 22]. This revealed that ever-drinking was significantly associated with a decreased risk of all-cause and breast cancer-specific death among the patients with ER- cancer (all-cause death, HR = 0.52, p = 0.04; breast cancer-specific death, HR = 0.43, p = 0.02 in comparison to never-drinking).

**Table 3:** Hazard ratio of all-cause and breast cancer-specific death associated with alcohol drinking by hormone receptor positive and negative type.

Factor	Patients	Person-years	All-cause death		Breast cancer-specific death	
			Deaths	HR (95% CI)^a^	Deaths	HR (95% CI)^a^
**ER+ or PR+**						
History of alcohol drinking						
Never	694	5572.9	105	1 (Reference)	67	1 (Reference)
Ever (Current/past)	286	2339.6	29	0.75 (0.47–1.21)	21	0.67 (0.38–1.18)
Past	31	244.8	2	0.49 (0.11–2.08)	2	0.90 (0.20–4.04)
Current	255	2094.8	27	0.78 (0.48–1.26)	19	0.65 (0.36–1.17)
Age at start of drinking (years)						
≥ 21	122	1015.1	14	0.64 (0.34–1.20)	10	0.62 (0.28–1.34)
≤ 20	142	1144.0	13	0.98 (0.51–1.89)	9	0.75 (0.34–1.65)
p for trend				0.56		0.28
Frequency of drinking (times)						
Occasional or 1–2 per week	136	1116.8	12	0.77 (0.41–1.48)	8	0.59 (0.27–1.31)
3–7 per week	137	1121.4	15	0.74 (0.40–1.37)	11	0.73 (0.34–1.53)
p for trend				0.28		0.26
Amount of alcohol consumed per day (g)						
< 5.0	123	1017.9	10	0.68 (0.34–1.35)	7	0.53 (0.23–1.22)
≥ 5.0	142	1162.3	15	0.71 (0.38–1.32)	11	0.69 (0.32–1.46)
p for trend				0.20		0.19
**ER-/PR-**						
History of alcohol drinking						
Never	242	1806.1	67	1 (Reference)	58	1 (Reference)
Ever (Current/past)	99	775.4	18	0.49 (0.26–0.94)	14	0.41 (0.20–0.84)
Past	13	81.4	4	0.43 (0.11–1.72)	2	0.15 (0.02–0.92)
Current	86	694.0	14	0.50 (0.25–1.00)	12	0.48 (0.23–1.00)
Age at start of drinking (years)						
≥ 21	43	343.7	8	0.44 (0.19–1.04)	7	0.46 (0.18–1.13)
≤ 20	47	364.3	9	0.71 (0.30–1.67)	6	0.44 (0.16–1.17)
p for trend				0.18		0.04
Frequency of drinking (times)						
Occasional or 1–2 per week	44	353.5	6	0.47 (0.19–1.16)	4	0.36 (0.12–1.08)
3–7 per week	50	392.7	9	0.45 (0.19–1.04)	8	0.45 (0.18–1.09)
p for trend				0.03		0.04
Amount of alcohol consumed per day (g)						
< 5.0	42	334.8	6	0.52 (0.20–1.31)	4	0.41 (0.13–1.24)
≥ 5.0	50	403.5	9	0.42 (0.18–0.96)	8	0.41 (0.17–0.99)
p for trend				0.03		0.03
p for interaction between ever-drinking*hormone receptor			0.47		0.20	

Abbreviations: HR, hazard ratio; CI, confidence interval; ER, estrogen receptor; PR, progesterone receptor.

^a^Adjusted by age, year of diagnosis, stage (0-I, II, III, IV, missing), referral status (from screening, other), radiation therapy (no, yes), chemotherapy (no, yes), endocrine therapy (no, yes), family history of breast cancer in parents or siblings (no, yes), smoking (ever, never, missing), BMI(<21.1, 21.1-<23.3, 23.3-<26.0, 26.0-, missing), physical activity (almost no, more than one hour per week, missing), comorbidities (no, yes), occupation (housewife, other, missing), age at menarche (≤12, 13, 14, 15<, missing), parity history (nulliparous, parous, missing), menopausal status (premenopausal, postmenopausal, missing), and intake of folate (continuous) and energy (continuous).

**Table 4:** Hazard ratio of all-cause and breast cancer-specific death associated with alcohol drinking by ER positive and negative type.

Factor	Patients	Person-years	All-cause death		Breast cancer-specific death	
			Deaths	HR (95% CI)^a^	Deaths	HR (95% CI)^a^
**ER+**						
History of alcohol drinking						
Never	676	5430.1	99	1 (Reference)	62	1 (Reference)
Ever (Current/past)	282	2300.2	29	0.80 (0.50–1.29)	21	0.75 (0.42–1.34)
Past	31	244.8	2	0.53 (0.12–2.26)	2	1.13 (0.25–4.99)
Current	251	2055.4	27	0.83 (0.51–1.36)	19	0.72 (0.39–1.31)
Age at start of drinking (years)						
≥ 21	121	1004.1	14	0.65 (0.34–1.23)	10	0.64 (0.29–1.44)
≤ 20	140	1126.7	13	1.12 (0.58–2.17)	9	0.91 (0.41–2.03)
p for trend				0.82		0.55
Frequency of drinking (times)						
Occasional or 1–2 per week	134	1099.4	12	0.81 (0.42–1.56)	8	0.64 (0.29–1.43)
3–7 per week	135	1099.4	15	0.80 (0.43–1.50)	11	0.86 (0.40–1.84)
p for trend				0.43		0.50
Amount of alcohol consumed per day (g)						
< 5.0	121	1000.6	10	0.71 (0.35–1.43)	7	0.57 (0.25–1.34)
≥ 5.0	140	1140.3	15	0.78 (0.42–1.47)	11	0.81 (0.38–1.76)
p for trend				0.34		0.40
**ER-**	** **	** **	** **	** **	** **	** **
History of alcohol drinking						
Never	260	1949.0	73	1 (Reference)	63	1 (Reference)
Ever (Current/past)	103	814.7	18	0.52 (0.27–0.98)	14	0.43 (0.21–0.87)
Past	13	81.4	4	0.50 (0.13–1.86)	2	0.19 (0.03–1.08)
Current	90	733.4	14	0.52 (0.26–1.04)	12	0.49 (0.24–1.03)
Age at start of drinking (years)						
≥ 21	44	354.7	8	0.53 (0.23–1.20)	7	0.55 (0.23–1.31)
≤ 20	49	381.7	9	0.69 (0.29–1.62)	6	0.40 (0.15–1.08)
p for trend				0.20		0.04
Frequency of drinking (times)						
Occasional or 1–2 per week	46	370.9	6	0.48 (0.20–1.20)	4	0.38 (0.13–1.11)
3–7 per week	52	414.7	9	0.48 (0.21–1.10)	8	0.45 (0.19–1.10)
p for trend				0.04		0.04
Amount of alcohol consumed per day (g)						
< 5.0	44	352.2	6	0.52 (0.21–1.31)	4	0.42 (0.14–1.24)
≥ 5.0	52	425.5	9	0.45 (0.20–1.03)	8	0.42 (0.18–1.02)
p for trend				0.04		0.03
p for interaction between ever-drinking*hormone receptor			0.41		0.19	

Abbreviations: HR, hazard ratio; CI, confidence interval; ER, estrogen receptor.

^a^Adjusted by age, year of diagnosis, stage (0-I, II, III, IV, missing), referral status (from screening, other), radiation therapy (no, yes), chemotherapy (no, yes), endocrine therapy (no, yes), family history of breast cancer in parents or siblings (no, yes), smoking (ever, never, missing), BMI(<21.1, 21.1-<23.3, 23.3-<26.0, 26.0-, missing), physical activity (almost no, more than one hour per week, missing), comorbidities (no, yes), occupation (housewife, other, missing), age at menarche (≤12, 13, 14, 15<, missing), parity history (nulliparous, parous, missing), menopausal status (premenopausal, postmenopausal, missing), and intake of folate (continuous) and energy (continuous).

Table 5 shows the results of analyses stratified by selected factors. Alcohol intake tended to reduce the risk of all-cause and breast cancer-specific death among premenopausal patient (all cause death, HR for ever-drinking = 0.54, p = 0.050; breast cancer-specific death, HR = 0.45, p = 0.02 in comparison to never-drinking). The analysis according to BMI level showed nonsignificant results. With regard to folate intake, a significantly decreased risk of death associated with alcohol consumption was observed in patients with low folate intake (all cause death, HR for ever-drinking = 0.58, p = 0.04; breast cancer-specific death, HR = 0.42, p = 0.01 in comparison to never-drinking)

**Table 5:** Hazard ratio of all-cause and breast cancer-specific death associated with alcohol drinking according to selected factors.

Factor	Patients	Person-years	All-cause death		Breast cancer-specific death	
			Deaths	HR (95% CI)	Deaths	HR (95% CI)
**Menopausal status**^**a**^						
**Premenupause**						
History of alcohol drinking						
Never	322	2612.7	52	1 (Reference)	48	1 (Reference)
Ever (Current/past)	218	1841.0	21	0.54 (0.29–1.00)	16	0.45 (0.23–0.89)
Amount of alcohol consumed per day (g)						
< 5.0	94	805.4	4	0.20 (0.06–0.63)	2	0.12 (0.02–0.54)
≥ 5.0	114	970.4	13	0.54 (0.26–1.10)	11	0.50 (0.23–1.09)
p for trend				0.05		0.04
**Postmenopause**						
History of alcohol drinking						
Never	648	4964.3	136	1 (Reference)	84	1 (Reference)
Ever (Current/past)	163	1212.3	29	0.92 (0.57–1.47)	23	0.85 (0.48–1.49)
Amount of alcohol consumed per day (g)						
< 5.0	65	491.4	13	1.06 (0.56–2.01)	10	0.92 (0.43–1.97)
≥ 5.0	80	588.2	14	0.89 (0.48–1.67)	12	0.91 (0.44–1.88)
p for trend				0.79		0.78
p for interaction between ever-drinking and menopausal status			0.82		0.47	
**BMI**^**b**^						
**<23.3 (low BMI)**						
History of alcohol drinking						
Never	452	3437.8	98	1 (Reference)	76	1 (Reference)
Ever (Current/past)	243	1987.2	35	0.77 (0.50–1.19)	25	0.61 (0.36–1.03)
Amount of alcohol consumed per day (g)						
< 5.0	103	849.4	14	0.79 (0.43–1.45)	9	0.54 (0.26–1.15)
≥ 5.0	126	1027.6	17	0.70 (0.39–1.26)	13	0.58 (0.29–1.16)
p for trend				0.19		0.06
**≥23.3 (high BMI)**						
History of alcohol drinking						
Never	551	4431.5	103	1 (Reference)	70	1 (Reference)
Ever (Current/past)	158	1246.8	19	0.81 (0.46–1.41)	17	0.91 (0.48–1.72)
Amount of alcohol consumed per day (g)						
< 5.0	67	544.7	5	0.47 (0.18–1.21)	5	0.55 (0.20–1.50)
≥ 5.0	75	599.5	11	1.04 (0.51–2.11)	10	1.22 (0.56–2.68)
p for trend				0.69		0.90
p for interaction between ever-drinking and body mass index			0.59		0.19	
**Folate intake**^**c**^						
**<207.8μg per day (low folate intake)**						
History of alcohol drinking						
Never	482	3597.4	104	1 (Reference)	84	1 (Reference)
Ever (Current/past)	228	1805.1	24	0.58 (0.35–0.98)	17	0.42 (0.23–0.78)
Amount of alcohol consumed per day (g)						
< 5.0	94	741.2	8	0.54 (0.25–1.17)	5	0.35 (0.13–0.91)
≥ 5.0	122	970.7	15	0.63 (0.33–1.19)	12	0.50 (0.24–1.04)
p for trend				0.09		0.02
**≥207.8μg per day (high folate intake)**						
History of alcohol drinking						
Never	533	4325.2	102	1 (Reference)	66	1 (Reference)
Ever (Current/past)	177	1453.1	31	1.08 (0.68–1.73)	26	1.20 (0.69–2.08)
Amount of alcohol consumed per day (g)						
< 5.0	77	654.7	12	0.94 (0.48–1.84)	10	1.00 (0.47–2.13)
≥ 5.0	82	678.7	13	1.06 (0.55–2.03)	11	1.27 (0.60–2.70)
p for trend				0.91		0.58
p for interaction between ever-drinking and folate intake			0.06		0.01	

Abbreviations: HR, hazard ratio; CI, confidence interval; ER, estrogen receptor; PR, progesterone receptor; BMI, body mass index.

^a^Adjusted by age, year of diagnosis, stage (0-I, II, III, IV, missing), hormone receptor (ER+ or PR+, ER-/PR-, missing), referral status (from screening, other), radiation therapy (no, yes), chemotherapy (no, yes), endocrine therapy (no, yes), family history of breast cancer in parents or siblings (no, yes), smoking (ever, never, missing), BMI(<21.1, 21.1-<23.3, 23.3-<26.0, 26.0-, missing), physical activity (almost no, more than one hour per week, missing), comorbidities (no, yes), occupation (housewife, other, missing), age at menarche (≤12, 13, 14, 15<, missing), parity history (nulliparous, parous, missing), and intake of folate (continuous) and energy (continuous).

^b^Adjusted by age, year of diagnosis, stage (0-I, II, III, IV, missing), hormone receptor (ER+ or PR+, ER-/PR-, missing), referral status (from screening, other), radiation therapy (no, yes), chemotherapy (no, yes), endocrine therapy (no, yes), family history of breast cancer in parents or siblings (no, yes), smoking (ever, never, missing), physical activity (almost no, more than one hour per week, missing), comorbidities (no, yes), occupation (housewife, other, missing), age at menarche (≤12, 13, 14, 15<, missing), parity history (nulliparous, parous, missing), menopausal status (premenopausal, postmenopausal, missing), and intake of folate (continuous) and energy (continuous).

^c^Adjusted by age, year of diagnosis, stage (0-I, II, III, IV, missing), hormone receptor (ER+ or PR+, ER-/PR-, missing), referral status (from screening, other), radiation therapy (no, yes), chemotherapy (no, yes), endocrine therapy (no, yes), family history of breast cancer in parents or siblings (no, yes), smoking (ever, never, missing), BMI(<21.1, 21.1-<23.3, 23.3-<26.0, 26.0-, missing), physical activity (almost no, more than one hour per week, missing), comorbidities (no, yes), occupation (housewife, other, missing), age at menarche (≤12, 13, 14, 15<, missing), parity history (nulliparous, parous, missing), menopausal status (premenopausal, postmenopausal, missing) and energy intake (continuous).

## Discussion

In this hospital-based patient cohort study, we clarified the association between pretreatment alcohol intake and the risk of all-cause and breast cancer-specific death among Japanese women with breast cancer. Ever-drinking tended to be associated with a decreased risk of all-cause and breast cancer-specific death. In terms to hormone receptor status, the decreased risk of death appeared to be limited to women with receptor-negative cancer. To our knowledge, our study is the first to have investigated the association of alcohol consumption with breast cancer survival in Asian women, whose alcohol intake is low relative to that of Caucasian women [21, 24]. The proportion of ever-drinkers among our study subjects was, in fact, quite low (28.5%).

Alcohol consumption has been considered an established risk factor for breast cancer. However, it has been unclear whether alcohol intake before breast cancer diagnosis affects the risk of mortality after diagnosis. One meta-analysis found that moderate drinkers had better overall survival, although it was not possible to estimate the combined risk for breast cancer-specific mortality because of the data heterogeneity among individual studies [22]. Another meta-analysis found no association between alcohol drinking and the risk of breast cancer-specific mortality, although analysis based on alcohol consumption level indicated increased mortality among heavy drinkers [23]. In the present study, a decreased risk of breast cancer-specific death was observed among ever-drinkers (HR = 0.68, 95% CI: 0.46–0.99, p = 0.047 in comparison to never-drinkers). The decreased risk was also found for light drinkers who drank occasionally or had <1–2 times per week (HR = 0.57, 95% CI: 0.33–0.99, p = 0.048), or an intake of <5.0 g of alcohol per day (HR = 0.57, 95% CI: 0.32–1.00, p = 0.050). For the risk of all-cause death, a non-linear association with alcohol consumption level was suggested. Based on these previous studies [13, 14, 16, 17, 20, 21] and ours, pre-diagnosis light to moderate alcohol consumption does not appear to have an adverse effect on survival among women with breast cancer. The effect of alcohol on the course of the disease may differ between breast cancer risk and survival after diagnosis.

Previous studies have demonstrated some health effects of alcohol consumption. Among community-dwelling women, light to moderate alcohol drinking was associated with a decreased risk of all-cause death [35, 36]. It has been suggested that this survival benefit may reflect a lower risk of death from heart disease among alcohol drinkers [35]. The decreased risk of death from cardiovascular disease associated with alcohol drinking has also been suggested in women with breast cancer [13, 14, 17]. None of the alcohol drinkers in the present series died of vascular diseases, as shown in Table 1, thus supporting the cardioprotective effect of alcohol. Besides, alcohol consumption might be related to psychosocial and physical status among breast cancer patients, thus explaining the improved overall and breast cancer-specific survival. Although excessive alcohol intake may be associated with poor health status [37, 38], light to moderate drinkers may have better physical and mental health and sociability than non-drinkers [37–41]. According to our previous population-based cohort study investigating the association of personality with breast cancer risk and survival, none of the personality subscales was associated with breast cancer risk, whereas subsequent follow-up of incident cases of breast cancer demonstrated that a higher score for extraversion, representing sociability and liveliness, tended to be associated with a lower risk of all-cause-death [42]. In that study, breast cancer patients with a higher score for extraversion tended to be ever-drinkers at the baseline [42], suggesting that interrelations between psychological factors and alcohol drinking may have some beneficial effects on survival.

In terms of hormone receptor status, the decreased risk of death associated with ever-drinking was observed among patients with receptor-negative cancer but not among those with receptor-positive cancer (all-cause death among ER-/PR- patients, HR for ever-drinking = 0.49, 95% CI: 0.26–0.94, p = 0.03; breast cancer-specific death among ER-/PR- patients, HR = 0.41, 95% CI: 0.20–0.84, p = 0.01 in comparison to never-drinking). Several previous studies have investigated the risk according to hormone receptor status, but the results have been inconsistent [13, 21, 22]. A decreased risk associated with pre-diagnosis alcohol drinking among patients with ER- cancer has been reported by the Women’s Health Initiative group [21]. The SEARCH study has also reported a decreased risk among ER- patients; however, that study evaluated the effects of post-diagnosis drinking [22]. With regard to the association of alcohol intake with survival of receptor-positive cancer, alcohol consumption is associated with increased endogenous estrogen levels, which may affect the disease progression and survival [43, 44]. The analysis in the subsample of our study subjects has also shown that intra-tumoral estradiol level in ever-drinkers with receptor-positive tumor tended to be higher than that in never-drinkers [45]. Furthermore, it is possible that alcohol may attenuate the anti-proliferative effects of endocrine therapeutic agents in women with ER+ cancer [46]. Therefore, alcohol consumption is unlikely to have a favorable effect among women with receptor-positive breast cancer. On the other hand, the mechanism responsible for the improved survival among women with receptor-negative cancer is unclear. One possibility is that the favorable effects of light to moderate alcohol drinking on metabolic and immune function, such as an anti-inflammatory effect, may protect against the development of receptor-negative cancer [47]. Furthermore, although hypothetical, genetic differences between drinkers and non-drinkers may modify the response of receptor-negative patients to chemotherapy. Subset of women who chose not to drink alcohol may be at a disadvantage, in view of their possibly reduced ability to metabolize chemotherapeutic agents and tendency to suffer emesis [20, 48].

Analyses stratified by other selected factors have found improved survival among premenopausal women who drink alcohol. In some previous studies, heavy drinking was associated with an increased risk of all-cause and breast cancer-specific death among postmenopausal patients [13, 14, 16]. As for premenopausal patients, one study including young patients (age ≤45 years) demonstrated improved survival associated with pre-diagnosis alcohol consumption [20]. Our present result appears to be comparable with this. In terms to folate intake, a lower risk of death was found among ever-drinkers who had a low folate intake. The modifiable effect of folate on alcohol-related breast cancer risk has been examined in a previous study, which demonstrated that the effect of alcohol intake on breast cancer risk may be reduced if the intake of folate is high [31]. On the other hand, few studies have evaluated the modifiable effect of folate on prognosis [14]. Our present study indicated that low folate intake appeared to have a beneficial effect on alcohol-related breast cancer survival, which was inconsistent with the impact of folate intake on breast cancer risk. Folate may exert either an anti- or pro-carcinogenic effect, depending on alcohol intake level and the timing of disease progression [20, 49–51].

The present study had both strengths and limitations. One of the strengths was a high quality of follow-up; only 3 patients (0.2%) were lost to follow-up. The quality of the clinical data was also high. For example, there were very few patients for whom hormone receptor status was unknown (Table 1). Another strength was that confounding factors including clinical factors and treatment were appropriately controlled for, and possible prognostic factors (reproductive and lifestyle factors) were also considered. The adjustment for these confounders seems to have improved internal validity of the present study. Among the study limitations, first, the history of alcohol drinking was self-reported. However, since the questionnaire survey was performed at the time of first admission before any definite diagnosis or treatment, any information bias would have been minimal. Second, since Japanese patients had a relatively low alcohol intake, it was not possible to fully investigate the mortality risk for heavy drinking. Third, although our study is one of the largest patient cohort studies of its kind to have been performed in Japan, stratification by hormone receptor status and other selected factors may have produced false positive or false negative results due to the small number of events. Breast cancer has a favorable prognosis, compared with other cancers. The overall survival rate in our study subjects was relatively high, which was similar to those from national reports in Japan [6, 7]. Although further studies including collaborative studies are required to confirm our results, the results provide clues for finding appropriate alcohol consumption levels in women. Finally, the present study did not collect information on changes in alcohol intake subsequent to cancer diagnosis and treatment. Therefore, we were unable to investigate the effect of post-diagnosis alcohol drinking.

In conclusion, the present patient cohort study of Japanese women with breast cancer has shown that pretreatment alcohol consumption was associated with a decreased risk of all-cause and breast cancer-specific death. According to alcohol consumption level, the decreased risk of breast cancer-specific death was found for light drinkers. In terms of hormone receptor status, a decreased risk of death associated with ever-drinking was observed among women with receptor-negative cancer but not among those with receptor-positive cancer. These findings suggest that pre-diagnosis alcohol intake is unlikely to have an adverse effect on survival after breast cancer diagnosis. Light alcohol consumption may have a beneficial effect on patient survival. However, alcohol drinking is associated with an increased risk of breast cancer. To improve breast cancer risk and survival, further studies will need to clarify the appropriate levels and timing of alcohol consumption.

## Supporting information

S1 File Questionnaire: English version. (PDF)

S2 File Questionnaire: Japanese version. (PDF)

## References

[1] Hamajima N, Hirose K, Tajima K, Rohan T, Calle EE, Heath CW Jr, et al Alcohol, tobacco and breast cancer-collaborative reanalysis of individual data from 53 epidemiological studies, including 58,515 women with breast cancer and 95,067 women without the disease. Br J Cancer. 2002;87: 1234–1245. 10.1038/sj.bjc.6600596 12439712 PMC2562507

[2] Key J, Hodgson S, Omar RZ, Jensen TK, Thompson SG, Boobis AR, et al Meta-analysis of studies of alcohol and breast cancer with consideration of the methodological issues. Cancer Causes Control. 2006;17: 759–770. 10.1007/s10552-006-0011-0 16783604

[3] Lin Y, Kikuchi S, Tamakoshi K, Wakai K, Kondo T, Niwa Y, et al Prospective study of alcohol consumption and breast cancer risk in Japanese women. Int J Cancer. 2005; 116: 779–783. 10.1002/ijc.20980 15838830

[4] Suzuki R, Iwasaki M, Inoue M, Sasazuki S, Sawada N, Yamaji T, et al; Japan Public Health Center-Based Prospective Study Group. Alcohol consumption-associated breast cancer incidence and potential effect modifiers: the Japan Public Health Center-based Prospective Study. Int J Cancer. 2010;127: 685–695. 10.1002/ijc.25079 19960437

[5] Allemani C, Matsuda T, Di Carlo V, Harewood R, Matz M, Nikšić M, et al; CONCORD Working Group. Global surveillance of trends in cancer survival 2000–14 (CONCORD-3): analysis of individual records for 37 513 025 patients diagnosed with one of 18 cancers from 322 population-based registries in 71 countries. Lancet. 2018;391 (10125): 1023–1075. 10.1016/S0140-6736(17)33326-3 29395269 PMC5879496

[6] Yoshimura A, Ito H, Nishino Y, Hattori M, Matsuda T, Miyashiro I, et al Recent improvement in the long-term survival of breast cancer patients by age and stage in Japan. J Epidemiol. 2018;28: 420–427. 10.2188/jea.JE20170103 29479003 PMC6143379

[7] Survival statistics of Japanese Association of Clinical Cancer Centers. Cancer survival rates at Japanese Association of Clinical Cancer Centers. Available from: https://kapweb.chiba-cancer-registry.org/?lang=en

[8] Katanoda K, Hori M, Matsuda T, Shibata A, Nishino Y, Hattori M, et al An updated report on the trends in cancer incidence and mortality in Japan, 1958–2013. Jpn J Clin Oncol. 2015;45: 390–401 10.1093/jjco/hyv002 25637502

[9] Rock CL, Doyle C, Demark-Wahnefried W, Meyerhardt J, Courneya KS, Schwartz AL, et al Nutrition and physical activity guidelines for cancer survivors. CA Cancer J Clin. 2012;62: 243–274. 10.3322/caac.21142 22539238

[10] Hebert JR, Hurley TG, Ma Y. The effect of dietary exposures on recurrence and mortality in early stage breast cancer. Breast Cancer Res Treat. 1998;51: 17–28. 10.1023/a:1006056915001 9877026

[11] Jain MG, Ferrenc RG, Rehm JT, Bondy SJ, Rohan TE, Ashley MJ, et al Alcohol and breast cancer mortality in a cohort study. Breast Cancer Res Treat. 2000;64: 201–209. 10.1023/a:1006402323445 11194456

[12] Allemani C, Berrino F, Krogh V, Sieri S, Pupa SM, Tagliabue E, et al Do pre-diagnostic drinking habits influence breast cancer survival? Tumori. 2011;97: 142–148. 10.1700/667.7774 21617706

[13] Vrieling A, Buck K, Heinz J, Obi N, Benner A, Flesch-Janys D, et al Pre-diagnostic alcohol consumption and postmenopausal breast cancer survival: a prospective patient cohort study. Breast Cancer Res Treat. 2012;136: 195–207. 10.1007/s10549-012-2230-2 22961011

[14] Harris HR, Bergkvist L, Wolk A. Alcohol intake and mortality among women with invasive breast cancer. Br J Cancer. 2012;106: 592–595. 10.1038/bjc.2011.561 22215064 PMC3273342

[15] Holm M, Olsen A, Christensen J, Kroman NT, Bidstrup PE, Johansen C, et al Pre-diagnostic alcohol consumption and breast cancer recurrence and mortality: results from a prospective cohort with a wide range of variation in alcohol intake. Int J Cancer. 2013;132: 686–694. 10.1002/ijc.27652 22623182

[16] Weaver AM, McCann SE, Nie J, Edge SB, Nochajski TH, Russell M, et al Alcohol intake over the life course and breast cancer survival in Western New York exposures and breast cancer (WEB) study: quantity and intensity of intake. Breast Cancer Res Treat. 2013;139: 245–253. 10.1007/s10549-013-2533-y 23605086 PMC3667502

[17] Newcomb PA, Kampman E, Trentham-Dietz A, Egan KM, Titus LJ, Baron JA, et al Alcohol consumption before and after breast cancer diagnosis: associations with survival from breast cancer, cardiovascular disease, and other causes. J Clin Oncol. 2013;31: 1939–1946. 10.1200/JCO.2012.46.5765 23569314 PMC3661933

[18] Zeinomar N, Thai A, Cloud AJ, McDonald JA, Liao Y, Terry MB. Alcohol consumption and breast cancer-specific and all-cause mortality in women diagnosed with breast cancer at the New York site of the Breast Cancer Family Registry. PLoS One. 2017 12 15;12(12):e0189118 10.1371/journal.pone.0189118 29244822 PMC5731703

[19] Parada H Jr, Sun X, Tse CK, Olshan AF, Troester MA. Lifestyle patterns and survival following breast cancer in the Carolina Breast Cancer Study. Epidemiology. 2019;30: 83–92. 10.1097/EDE.0000000000000933 30299404 PMC7451223

[20] Reding KW, Daling JR, Doody DR, O'Brien CA, Porter PL, Malone KE. Effect of prediagnostic alcohol consumption on survival after breast cancer in young women. Cancer Epidemiol Biomarkers Prev. 2008;17: 1988–1996. 10.1158/1055-9965.EPI-07-2897 18664549 PMC2605937

[21] Lowry SJ, Kapphahn K, Chlebowski R, Li CI. Alcohol Use and Breast Cancer Survival among Participants in the Women's Health Initiative. Cancer Epidemiol Biomarkers Prev. 2016;25: 1268–1273. 10.1158/1055-9965.EPI-16-0151 27197280 PMC4970888

[22] Ali AM, Schmidt MK, Bolla MK, Wang Q, Gago-Dominguez M, Castelao JE, et al Alcohol consumption and survival after a breast cancer diagnosis: a literature-based meta-analysis and collaborative analysis of data for 29,239 cases. Cancer Epidemiol Biomarkers Prev. 2014;23: 934–945. 10.1158/1055-9965.EPI-13-0901 24636975 PMC4542077

[23] Gou YJ, Xie DX, Yang KH, Liu YL, Zhang JH, Li B, et al Alcohol consumption and breast cancer survival: a meta- analysis of cohort studies. Asian Pac J Cancer Prev. 2013;14: 4785–4790. 10.7314/apjcp.2013.14.8.4785 24083744

[24] WHO. Global status report on alcohol and health 2014. Available from: https://www.who.int/substance_abuse/publications/alcohol_2014/en.html. Cited 16 May 2019.

[25] Kawai M, Minami Y, Nishino Y, Fukamachi K, Ohuchi N, Kakugawa Y. Body mass index and survival after breast cancer diagnosis in Japanese women. BMC Cancer. 2012;12: 149 10.1186/1471-2407-12-149 22510365 PMC3444378

[26] Kakugawa Y, Kawai M, Nishino Y, Fukamachi K, Ishida T, Ohuchi N, et al Smoking and survival after breast cancer diagnosis in Japanese women: A prospective cohort study. Cancer Sci. 2015;106: 1066–1074. 10.1111/cas.12716 26052951 PMC4556397

[27] Ogawa K, Tsubono Y, Nishino Y, Watanabe Y, Ohkubo T, Watanabe T, et al Validation of a food-frequency questionnaire for cohort studies in rural Japan. Public Health Nutr. 2003;6: 147–157. 10.1079/PHN2002411 12675957

[28] Kawai M, Minami Y, Kakizaki M, Kakugawa Y, Nishino Y, Fukao A, et al Alcohol consumption and breast cancer risk in Japanese women: the Miyagi Cohort study. Breast Cancer Res. Treat. 2011;128: 817–825. 10.1007/s10549-011-1381-x 21318600

[29] Takizawa Y, Kawai M, Kakugawa Y, Nishino Y, Ohuchi N, Minami Y. Alcohol Consumption and Breast Cancer Risk According to Hormone Receptor Status in Japanese Women: A Case-Control Study. Tohoku J Exp Med. 2018;244: 63–73. 10.1620/tjem.244.63 29353824

[30] Minami Y, Nishino Y, Kawai M, Tada H, Kanemura S, Miyashita M, et al Reproductive history and breast cancer survival: a prospective patient cohort study in Japan. Breast Cancer. (in press) 10.1007/s12282-019-00972-5 30993643

[31] Chen P, Li C, Li X, Li J, Chu R, Wang H. Higher dietary folate intake reduces the breast cancer risk: a systematic review and meta-analysis. Br J Cancer. 2014;10: 2327–2338. 10.1038/bjc.2014.155 PMC4007237 24667649

[32] Xie J, Liu C. Adjusted Kaplan-Meier estimator and log-rank test with inverse probability of treatment weighting for survival data. Stat Med. 2005;24:3089–3110. 10.1002/sim.2174 16189810

[33] Rothman KJ, Greenland S, Lash TL. Bias analysis In: Modern Epidemiology. 3rd ed Philadelphia: Lippincott Williams & Wilkins; 2008 pp. 345–380.

[34] Wan S, Lai Y, Myers RE, Li B, Palazzo JP, Burkart AL, et al Post-diagnosis hemoglobin change associates with overall survival of multiple malignancies—results from a 14-year hospital-based cohort of lung, breast, colorectal, and liver cancers. BMC Cancer. 2013;13: 340 10.1186/1471-2407-13-340 23841898 PMC3710492

[35] Fuchs CS, Stampfer MJ, Colditz GA, Giovannucci EL, Manson JE, Kawachi I, et al Alcohol consumption and mortality among women. N Engl J Med. 1995;332(19): 1245–1250. 10.1056/NEJM199505113321901 7708067

[36] Saito E, Inoue M, Sawada N, Charvat H, Shimazu T, Yamaji T, et al Impact of alcohol intake and drinking patterns on mortality from all causes and major causes of death in a Japanese population. J Epidemiol. 2018;28: 140–148. 10.2188/jea.JE20160200 29129895 PMC5821691

[37] Stranges S, Notaro J, Freudenheim JL, Calogero RM, Muti P, Farinaro E, et al Alcohol drinking pattern and subjective health in a population-based study. Addiction. 2006;101: 1265–1276. 10.1111/j.1360-0443.2006.01517.x 16911725

[38] Scott RG, Wiener CH, Paulson D. The benefit of moderate alcohol use on mood and functional ability in later life: due to beers or frequent cheers? Gerontologist. 2018 10 24 10.1093/geront/gny129 30358833

[39] Green CA, Perrin NA, Polen MR. Gender differences in the relationships between multiple measures of alcohol consumption and physical and mental health. Alcohol Clin Exp Res. 2004;28: 754–764. 10.1097/01.alc.0000125342.28367.a1 15166650

[40] Chan AM, von Mühlen D, Kritz-Silverstein D, Barrett-Connor E. Regular alcohol consumption is associated with increasing quality of life and mood in older men and women: the Rancho Bernardo Study. Maturitas. 2009;62: 294–300. 10.1016/j.maturitas.2009.01.005 19232847 PMC2681249

[41] Valencia-Martín JL, Galán I, Guallar-Castillón P, Rodríguez-Artalejo F. Alcohol drinking patterns and health-related quality of life reported in the Spanish adult population. Prev Med. 2013;57: 703–707. 10.1016/j.ypmed.2013.09.007 24051265

[42] Minami Y, Hosokawa T, Nakaya N, Sugawara Y, Nishino Y, Kakugawa Y, et al Personality and breast cancer risk and survival: the Miyagi cohort study. Breast Cancer Res Treat. 2015;150: 675–684. 10.1007/s10549-015-3364-9 25829230

[43] Dorgan JF, Baer DJ, Albert PS, Judd JT, Brown ED, Corle DK, et al Serum hormones and the alcohol-breast cancer association in postmenopausal women. J Natl Cancer Inst. 2001;93: 710–715. 10.1093/jnci/93.9.710 11333294

[44] Singletary KW, Gapstur SM. Alcohol and breast cancer: review of epidemiologic and experimental evidence and potential mechanisms. JAMA. 2001;286: 2143–2151. 10.1001/jama.286.17.2143 11694156

[45] Kakugawa Y, Tada H, Kawai M, Suzuki T, Nishino Y, Kanemura S, et al Associations of obesity and physical activity with serum and intratumoral sex steroid hormone levels among postmenopausal women with breast cancer: analysis of paired serum and tumor tissue samples. Breast Cancer Res Treat. 2017;162: 115–125. 10.1007/s10549-016-4094-3 28044214

[46] Candelaria NR, Weldon R, Muthusamy S, Nguyen-Vu T, Addanki S, Yoffou PH, et al Alcohol Regulates Genes that Are Associated with Response to Endocrine Therapy and Attenuates the Actions of Tamoxifen in Breast Cancer Cells. PLoS One. 2015 12 14;10(12):e0145061 10.1371/journal.pone.0145061 26661278 PMC4681367

[47] Romeo J, Wärnberg J, Nova E, Díaz LE, Gómez-Martinez S, Marcos A. Moderate alcohol consumption and the immune system: a review. Br J Nutr. 2007;98 Suppl 1: S111–S115. 17922947 10.1017/S0007114507838049

[48] Uomori T, Horimoto Y, Mogushi K, Matsuoka J, Saito M. Relationship between alcohol metabolism and chemotherapy-induced emetic events in breast cancer patients. Breast Cancer. 2017;24: 702–707. 10.1007/s12282-017-0761-4 28217830

[49] Kim YI. Will mandatory folic acid fortification prevent or promote cancer? Am J Clin Nutr. 2004;80: 1123–1128. 10.1093/ajcn/80.5.1123 15531657

[50] Wei EK, Wolin KY, Colditz GA. Time course of risk factors in cancer etiology and progression. J Clin Oncol. 2010;28: 4052–4057. 10.1200/JCO.2009.26.9324 20644083 PMC4872328

[51] Christensen BC, Kelsey KT, Zheng S, Houseman EA, Marsit CJ, Wrensch MR, et al Breast cancer DNA methylation profiles are associated with tumor size and alcohol and folate intake. PLoS Genet. 2010 7 29;6(7):e1001043 10.1371/journal.pgen.1001043 20686660 PMC2912395

