# Supplementary material for: Alcohol consumption and survival after breast cancer diagnosis in Japanese women: A prospective patient cohort study
Source: PLoS One. 2019 Nov 13;14(11):e0224797. doi: 10.1371/journal.pone.0224797 (PMC6853331; doi:10.1371/journal.pone.0224797)
Supplement: S1 File — (PDF) [file pone.0224797.s001.pdf]

(7) Are you able to toilet activities (e.g. change underwear, treatment of excrement) by yourself?
i. Yes, I can ii. Yes, with assistance iii. No, I can't

(8) Do you currently smoke cigarettes?Yes (current smoker)
1. Quitted smoking
(If so, circle the reason: i.because of illness ii.for health iii.others)
2. No (never smoker)
If you circled 1 or 2,

How old were you when you began smoking? (years old)
How many cigarettes per day did you smoke? (cigarettes per day)

(9) If you have been married, does your spouse smoke cigarettes?
1. Yes (currently smoking)
2. Quitted smoking (years ago,   month ago)
3. No (never smoking)

(10) Do you drink alcoholic beverages?
1. Yes
2. Quitted drinking (years ago,   month ago)
(If so, circle the reason: i.because of illness ii.for health iii.others)
3. No
If you circled 1 or 2,
1) How old were you when you began drinking alcohol beverages? (years old)
2) How often do (did) you drink alcohol beverages?
i. Almost every day (more than five times per week)
ii. Three to four times per week iii. One to two times per week
iv. Less than once a week

3) What kind of alcohol beverages do you often drink? Circle all apply.
i. Japanese sake ii. Shochu iii. Beer iv. Whisky v. Wine v. Others

4) How much do you drink alcohol beverage per one time? Convert into the amount of Japanese sake. (Have a look at the table below to calculate)
i. Less than one gou ii. About one gou iii. About two gou iv. About three gou
v. About four gou vi. More than five gou

One gou of shochu	=	1 ½ gou of Japanese sake
A large bottle of beer	=	1 gou of Japanese sake
A glass of Whisky (double)	=	1 gou of Japanese sake
A glass of wine	=	1 gou of Japanese sake
If you would like to mix and drink the alcohol beverages with water, calculate without amount of water.		
