# Supplementary material for: Alcohol consumption and survival after breast cancer diagnosis in Japanese women: A prospective patient cohort study
Source: PLoS One. 2019 Nov 13;14(11):e0224797. doi: 10.1371/journal.pone.0224797 (PMC6853331; doi:10.1371/journal.pone.0224797)
Supplement: S2 File — (PDF) [file pone.0224797.s002.pdf]

（7）トイレ動作（服，下着の上げ下げ，排泄後の後始末など）はできますか。

1．できる　2．介助されればできる　3．できない

（8）あなたはタバコを吸いますか。

1．吸っている　2．止めた（　　年前，　　ケ月前）　3．吸わない

└→ **止めた理由**　ア．病気のため　イ．健康保持のため　ウ．その他

1または2に○をつけた場合，

（　　　）歳から吸い始め，1日に平均（　　　）本くらい

（9）結婚されている場合，あなたの配偶者（夫または妻）はタバコを吸っておられますか。

1．吸っている　2．止めた（　　年前，　　ケ月前）　3．吸わない

（10）あなたはお酒（アルコール類）を飲みますか。

1．飲む　2．止めた（　　年前，　　ケ月前）　3．ほとんど飲まない

└→ **止めた理由**　ア．病気のため　イ．健康保持のため　ウ．その他

1または2に○をつけた場合，

1）飲み始めたのは何歳の時ですか。　（　　　）歳

2）どれ位飲みますか（飲んでいましたか）。

ア．ほとんど毎日（週5回以上）　イ．週3～4回

ウ．週1～2回　エ．週1回以下

3）あなたがふつうよく飲む酒に○をつけて下さい。（いくつでも）

ア．日本酒　イ．焼酎　ウ．ビール　エ．ウイスキー

オ．ワイン　カ．その他

4）飲む量は平均すると，1回あたりどの位ですか。日本酒に換算してください。（換算のし方は，表を参照してください）

ア．1合より少ない　イ．約1合　ウ．約2合

エ．約3合　オ．約4合　カ．約5合以上

焼酎1合は ………………	日本酒約1.5合	に当たる
ビール大びん1本は ………………	日本酒約1合	
ウイスキーダブル1杯は ………………	日本酒約1合	
ワイン1合は ………………	日本酒約1合	

うすめて飲むときは，うすめる前の量で計算してください
